# Neuroendocrine mechanisms responsible for elevated gonadotrophin‐releasing hormone and luteinising hormone pulses in polycystic ovary syndrome

**DOI:** 10.1111/jne.70028

**Published:** 2025-04-18

**Authors:** Aleisha M. Moore

**Affiliations:** ^1^ Department of Biological Sciences, Brain Health Research Institute Kent State University Kent Ohio USA

**Keywords:** dynorphin, gonadotrophin‐releasing hormone neuron, kisspeptin, luteinizing hormone, neurokinin B

## Abstract

Polycystic ovary syndrome (PCOS) is the leading cause of anovulatory infertility in premenopausal individuals with ovaries worldwide. Despite the diagnostic features of anovulation, ovarian cysts, and hyperandrogenemia, which indicate that ovary dysfunction is the cause of the syndrome, changes in central neuroendocrine circuits are a significant cause of PCOS pathology. Specifically, cells in the hypothalamus have a diminished ability to transmit negative feedback signals from gonadal sex steroid hormones to gonadotropin‐releasing hormone (GnRH) neurons. This results in an elevated frequency of pulsatile hypothalamic GnRH and pituitary luteinizing hormone (LH) secretion, leading to ovarian hyperandrogenism and ovulatory dysfunction. In recent years, preclinical research in animal models has rapidly advanced our understanding of the neural mechanisms underlying GnRH pulse generation with the identification of KNDy cells—a unique cell population in the hypothalamus expressing the neuropeptides kisspeptin, neurokinin B and dynorphin. As a result, therapeutics targeting KNDy cell signaling have emerged as a promising avenue for treating GnRH/LH hypersecretion in PCOS patients. However, the precise central changes underpinning impaired negative feedback regulation of GnRH pulse generation in PCOS patients are still unclear. Evidence from both the clinic and animal models suggests that changes in the regulation of KNDy cells may be directly responsible for elevated GnRH and LH pulse frequency in PCOS. However, other cell populations regulating GnRH secretion may also be involved. This review provides an overview of our current understanding of the aetiology and contribution of neuroendocrine dysfunction in PCOS pathology. It also examines the evidence for neural mechanisms underlying GnRH/LH hypersecretion, which may serve as central targets in developing novel treatments. Finally, this review highlights key knowledge gaps that are hindering the development of preventive and curative interventions.

## 1 INTRODUCTION

Polycystic ovary syndrome (PCOS) is the most common cause of anovulatory infertility and hyperandrogenism in reproductive‐aged individuals with ovaries.^1^ Although multiple criteria have been used in the diagnosis of PCOS,^2^ recent evidence‐based updates for the International Guidelines for the Assessment and Management of PCOS favor the Rotterdam Criteria,^3^ initially put forward in 2003 and requiring at least two out of three of the following conditions: irregular or absent menstrual cycles, evidence of increased androgen levels, and polycystic or poly‐follicular ovary morphology (PCOM) observed via ultrasound.^4^ In addition, elevated anti‐Müllerian hormone (AMH), secreted by granulosa cells of ovarian follicles, has recently been added as an alternative criterion to PCOM, avoiding the use of invasive ultrasounds during diagnosis.^5^ The prevalence of PCOS diagnosis with the Rotterdam Criteria can reach over 15%.^6^ The syndrome is further associated with major comorbidities, including type 2 diabetes, obesity, cardiovascular disease, gynecological cancers, eating disorders, sexual dysfunction, poorer performance on cognitive tasks, anxiety, and depression.^7^, ^8^, ^9^, ^10^, ^11^, ^12^ The clinical diversity of PCOS has made it historically challenging to develop treatments, and current management focuses on addressing symptoms rather than understanding the underlying causes of the syndrome.^13^ However, since the 1970s, changes within central networks regulating the pulsatile release of gonadotrophin‐releasing hormone (GnRH) from neurons in the hypothalamus have been regarded as central to the pathogenesis of PCOS.^14^ This review first provides an overview of the evidence that abnormal GnRH pulse generation is a significant cause of ovarian dysfunction in PCOS and a crucial target for treatment. The review then describes the current state of knowledge regarding the neural components and mechanisms contributing to abnormal GnRH pulse generation. Importantly, this section will highlight the crucial role that basic research in preclinical models has played in identifying the neural components underlying pulse generation, which have served as the basis for developing novel treatments aimed at improving pulse generation in PCOS patients. Finally, this review discusses some of the current challenges associated with the prevention and treatment of PCOS, highlighting the ongoing need to characterize altered circuitry that may contribute to reproductive and neuroendocrine symptoms, as well as associated comorbidities occurring in the syndrome.

## 2 NEUROENDOCRINE AETIOLOGY OF PCOS

PCOS is associated with a highly variable and complex combination of genetic, epigenetic, and environmental causes.^15^ The syndrome is heritable, as recently evidenced by a large register‐based study finding that daughters of PCOS patients have a fivefold increase in the risk of being diagnosed with the syndrome compared to daughters of people without PCOS.^16^ Although genetic loci linked to PCOS have been recognized as important contributors to the condition, they currently account for only about 10% of the estimated 70% heritability of the syndrome.^17^ Consequently, environmental and epigenetic mechanisms are thought to play a significant role in the remaining inheritance of PCOS. In particular, changes within the maternal‐fetal environment are anticipated to contribute to the inheritance of PCOS or create a favorable setting for gene variants that increase the risk of developing the syndrome. Prenatal insults during critical time windows of fetal development are widely associated with the risk of disease in later life, a concept known as the Barker hypothesis.^18^ In pregnant patients with PCOS, high levels of testosterone persist in pregnancy (reference 19 and reviewed in 20). These high testosterone levels have been found in the fetal environment, as measured in umbilical vein blood^21^ and amniotic fluid.^22^ PCOS daughters are also born with elongated anogenital distances and enhanced sebum production at birth, both indicating prenatal exposure to androgens and serving as a potential early biomarker for PCOS risk.^23^, ^24^ Exogenous delivery of androgens to pregnant animal models recapitulates the elongated anogenital distance as well as acyclicity, infertility, and altered ovarian morphology.^25^ The source of fetal androgen excess remains unclear but may be multifactorial. Variations in genes that encode androgen biosynthesis may contribute to either fetal or maternal androgen excess.^26^ Although placental aromatase usually prevents the latter from being passed into fetal circulation, the Giacobini group recently proposed a mechanism wherein maternal AMH, which is elevated in pregnant people with PCOS,^27^ stimulates luteinizing hormone (LH)‐dependent androgen secretion in the maternal circulation, which is transmitted to the fetus through the downregulation of placental aromatase activity.^27^ An increase in androgen exposure during prenatal life may also encode transgenerational inheritance of PCOS via epigenetic mechanisms, as mice that receive prenatal exposure to either dihydrotestosterone (referred to as prenatal androgen‐treated (PNA) mice) or prenatal AMH (PAMH) transmit reproductive traits to third‐generation female offspring.^16^, ^28^

**FIGURE 1:**
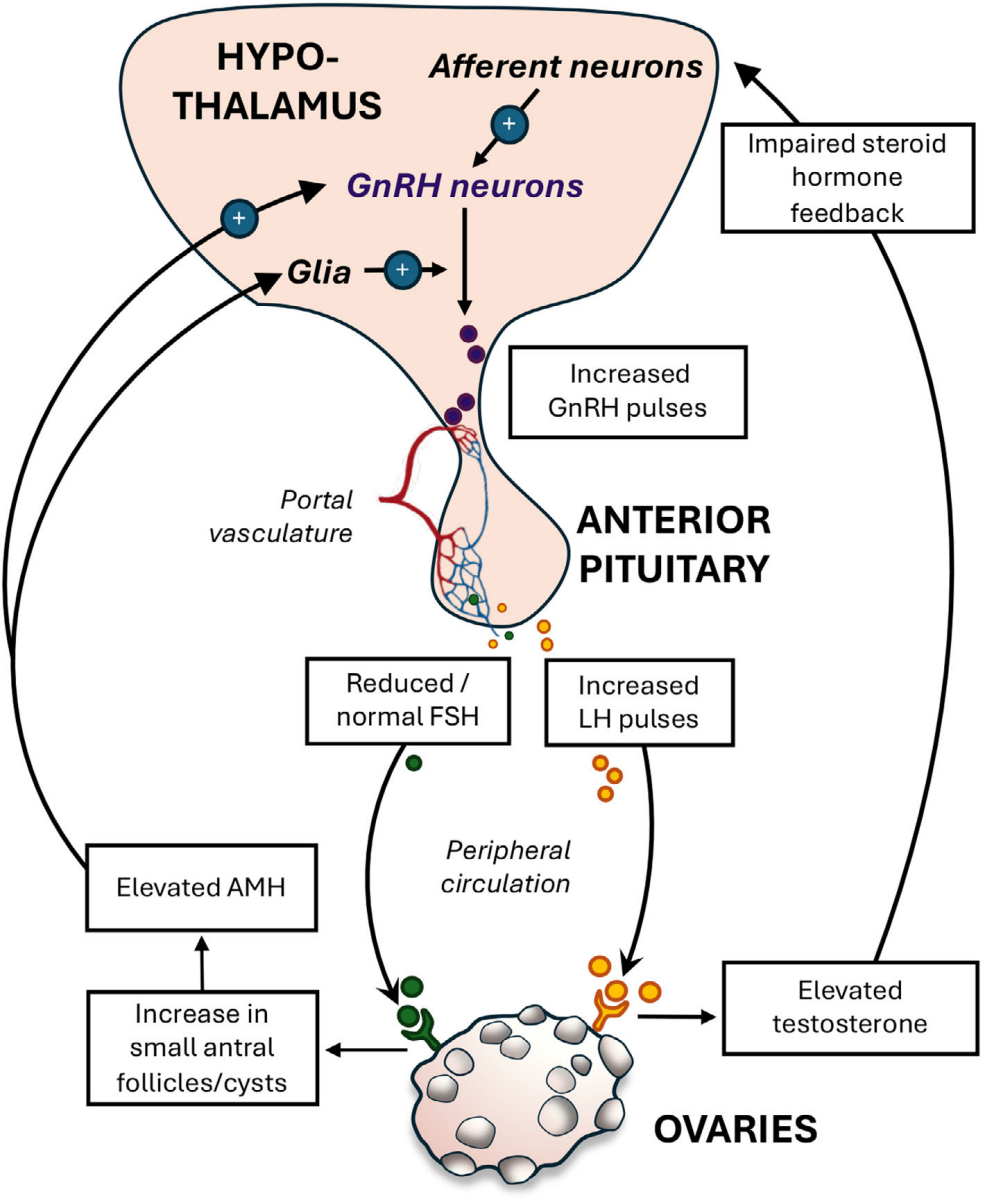
The hypothalamic–pituitary–gonadal axis in PCOS. PCOS has been dubbed a vicious cycle of pulsatile GnRH/LH and androgen release. In this cycle, elevated pulsatile LH secretion and reduced or normal FSH secretion result from elevated GnRH pulse frequency. At the ovary, reduced FSH signaling impairs antral follicle maturation, leading to the accumulation of small immature follicles and an increase in AMH secretion. LH hypersecretion drives the secretion of ovarian testosterone. At the brain, testosterone impairs the ability of upstream cells to relay negative feedback information to GnRH neurons, increasing GnRH secretion. Concomitantly, AMH may increase GnRH secretion either directly at GnRH neurons or via specialized glial cells that regulate the access of GnRH terminals to the pericapillary space.

In PCOS patients, once hyperandrogenism is established after puberty, the excessive secretion of ovarian androgens plays a central role in driving PCOS and is positively associated with the severity of reproductive and metabolic dysfunction.^29^ The trait is present in approximately 75%–90% of people diagnosed with the syndrome.^30^ In addition to evidence for intrinsic alterations within androgen‐secreting thecal cells,^31^ ovarian androgen hypersecretion is driven by LH (Figure 1). Over 75% of anovulatory PCOS patients have elevated serum LH concentrations, while the ratio of high LH to low FSH is detected in 94% of the tested population.^30^ As a result of reduced FSH, there is insufficient growth and maturation of follicles, resulting in anovulation.^30^ This elevated ratio results from an increase in the frequency of LH pulses from the anterior pituitary gland,^32^ representing an increase in pulsatile GnRH release from GnRH neurons within the hypothalamus. High LH pulse frequency is a consistent finding in PCOS regardless of obesity status, although the amplitude of LH pulses is suppressed in individuals with a high BMI.^33^ This may be the consequence of elevated metabolic hormones such as insulin desensitizing the pituitary to GnRH, as LH release in response to GnRH administration is attenuated in obese PCOS patients.^34^ Alternatively, metabolic or inflammatory factors may act at the level of the hypothalamus to blunt the pulsatile secretion of GnRH.^35^

In a more recent study, chronic overactivation of GnRH neurons and elevated LH pulsatility in normoandrogenic mice demonstrated that a “neuroendocrine trigger” is sufficient to induce hyperandrogenemia, PCO morphology, and acyclicity.^27^, ^36^ An additional contributing factor to the hyperandrogenemic state is hyperinsulinemia resulting from insulin resistance, which upregulates LH receptors and promotes androgen secretion at thecal cells.^37^ The biological importance of altered gonadotrophin secretion patterns is supported by genetic studies that detect variations in several genes for the LH and FSH subunits and the LH/choriogonadotrophin receptor in PCOS (reviewed in 38). LH‐induced hyperandrogenemia also explains how the use of long‐term GnRH agonist oral contraceptives to blunt gonadotrophin levels (due to pituitary desensitization) can reverse hyperandrogenism in PCOS.^39^

Notably, elevated LH pulse frequency in PCOS can be attributed to a diminished sensitivity toward estradiol and progesterone negative feedback mechanisms. In patients with normal fertility, ovarian estradiol and progesterone act in the hypothalamus at cells upstream from GnRH neurons to reduce GnRH/LH secretion. However, in PCOS patients, gonadal steroid hormone treatment fails to suppress GnRH/LH pulse frequency to the same extent as controls^40^ (Figure 1). Studies show that while estradiol and progesterone treatment reduce LH pulses by approximately 60% in healthy patients, the reduction is only 25% in people with PCOS. This resistance to negative feedback is partly due to the central impact of hyperandrogenism, as blocking androgen receptors with the antagonist Flutamide can restore normal feedback mechanisms.^41^ Therefore, PCOS represents a vicious cycle by which elevated GnRH/LH pulse frequency drives hyperandrogenemia, which can then act back in the brain to impair homeostatic negative feedback mechanisms. Importantly, changes in steroid hormone sensitivity are hypothesized to play a role as the primary trigger of PCOS during puberty. Elevated LH is detected during adolescence,^42^ suggesting it is a trait programmed in early development. In a model by McCartney and colleagues,^43^ GnRH pulse generation increases at the start of puberty and is dependent upon progesterone‐sensitive neuronal inputs suppressing GnRH/LH pulses during the daytime and progesterone‐insensitive inputs permitting high frequency at nighttime. In hyperandrogenemic girls, a loss of progesterone sensitivity during the day results in high‐frequency GnRH pulses during both sleep and wake phases, driving further ovarian androgen synthesis and solidifying the cycle of PCOS that interplays between ovaries and the brain. This indicates that a failure of progesterone to reduce GnRH/LH pulses leads to the establishment of excessive LH and inadequate production of FSH over puberty.

In addition to the interference of steroid hormone feedback by androgens, there is evidence that AMH, which is produced by ovarian follicles and correlated with the number of diagnostic features exhibited by a patient,^44^, ^45^, ^46^ can directly drive LH hypersecretion (Figure 1). Human GnRH neurons contain the receptor for AMH (AMH2R), and in adult rodents, AMH acts via this receptor to directly promote the excitation of GnRH neurons and drive elevated pulsatile LH release.^47^ It is therefore possible that AMH‐induced LH hypersecretion contributes to hyperandrogenism and may stimulate further AMH secretion,^36^ inducing a positive feedback loop between androgens, AMH, and GnRH/LH. However, further research is needed to evaluate the role of AMH in sustaining LH hypersecretion in patients with PCOS. While anti‐androgens can restore negative feedback,^41^ it remains untested whether AMH antagonism can reduce LH and testosterone levels in PCOS patients and animal models.

To conclude, PCOS is a syndrome that develops early during critical windows of development and creates a self‐perpetuating cycle with GnRH/LH, ovarian testosterone, and potentially AMH promoting the secretion of one another. Once this pattern is established, there is no known cure. Identifying the exact neural targets is essential for understanding the mechanisms behind increased GnRH pulse generation in PCOS and could highlight key areas for treatment.

## 3 CELL POPULATIONS MEDIATING ELEVATED GnRH PULSATILE SECRETION IN PCOS

**FIGURE 2:**
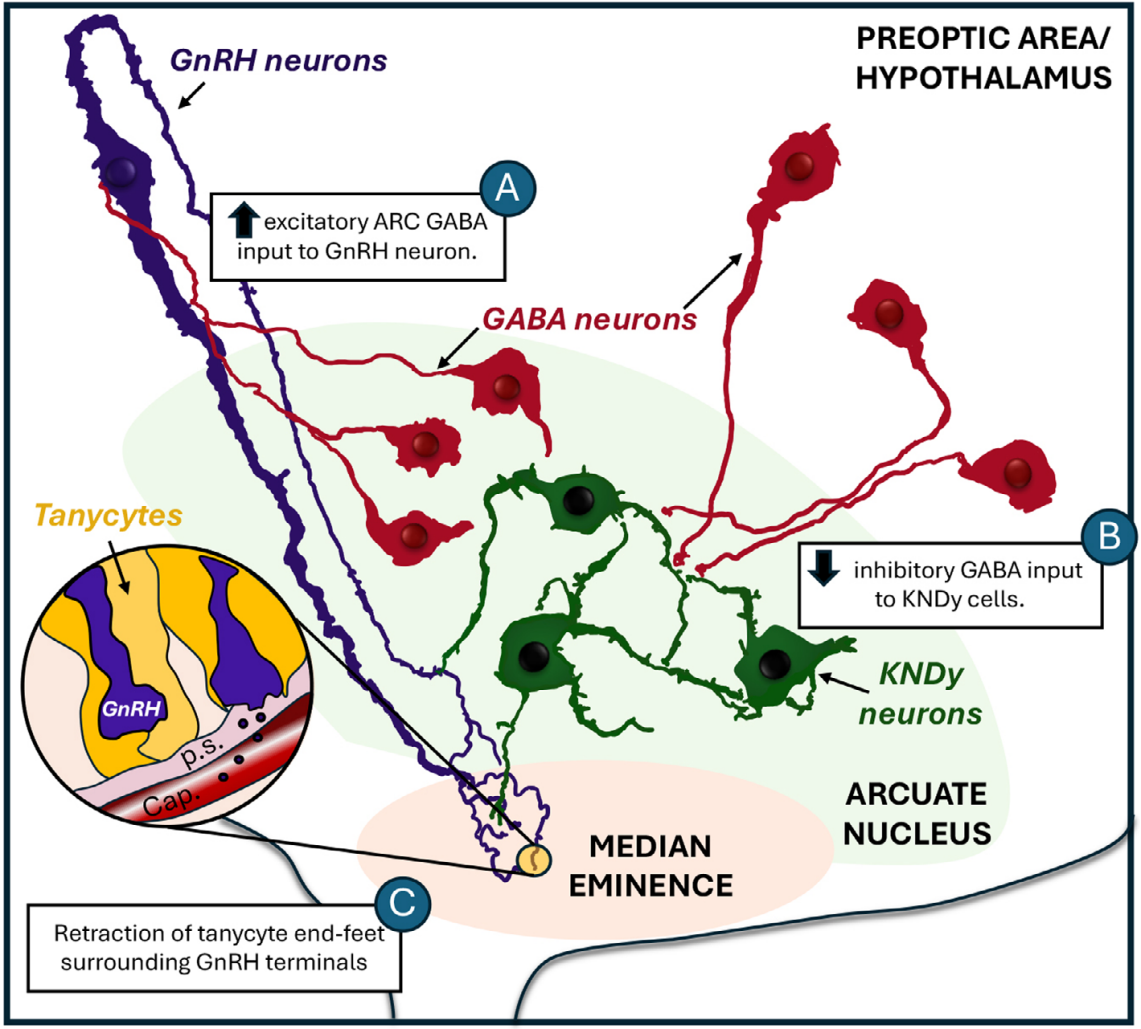
Structural plasticity within the GnRH neuronal network induced by elevated androgens and AMH in PCOS. Research using animal models generated with prenatal androgen or prenatal AMH exposure has revealed mechanisms via which these hormones promote GnRH secretion in PCOS: (A) Hyperandrogenemia increases excitatory GABAergic input to GnRH neurons, resulting in elevated GnRH secretion. This is associated with reduced pruning of GABA terminals to GnRH neurons by microglia during prenatal development. (B) Hyperandrogenemia reduces synaptic input from hypothalamic GABA cells to KNDy cells in the arcuate, a population that acts as the GnRH pulse generator. This may increase episodic activity in KNDy and GnRH neurons, resulting in elevated GnRH pulse frequency. (C) AMH induces the rapid retraction of tanycytic end‐feet around GnRH terminals in the median eminence, allowing direct contact of GnRH terminals with pericapillary space and facilitating the diffusion of GnRH into portal vasculature.

Several mechanistic changes in the brain have been associated with the increased pulsatile release of GnRH and LH in PCOS that appears after puberty. As it is not possible to study in vivo pathology within hypothalamic circuits of PCOS patients, most studies into the mechanistic underpinnings of GnRH hypersecretion have been conducted in non‐human primate, sheep, and rodent models of PCOS induced by prenatal androgen or AMH exposure (reviewed in 25). As GnRH neurons do not express gonadal steroid hormone receptors, the site of androgen interference with steroid hormone feedback is likely within afferent populations. In recent years, cells within the arcuate nucleus of the mediobasal hypothalamus, historically hypothesized to be the site of the GnRH pulse generator, have been primarily implicated (Figure 2).

### 3.1 GABA cells

The neurotransmitter GABA is a major regulator of GnRH neuron activity. Unlike in most adult neurons, the activation of GABA‐A receptors on GnRH neurons is excitatory due to a high intracellular chloride concentration within the cells.^48^ GABA levels in the cerebrospinal fluid and blood are significantly elevated in PCOS patients compared to controls, implicating a role in the pathophysiology of the syndrome.^49^, ^50^, ^51^ However, these circuits cannot be directly assessed in PCOS patients; instead, a large body of work has investigated this population using mouse and sheep models.^52^ In PNA and PAMH mice, there is an increase in excitatory GABAergic neurotransmission and innervation to the GnRH soma and rostral dendrite originating from the arcuate nucleus^27^, ^53^, ^54^ (Figure 2A). This was further evidenced in prenatal testosterone‐treated (PNT) sheep, in which an increase in GABAergic input was quantified to GnRH neurons in the MBH,^55^ a population specifically implicated in pulse secretion in both humans and sheep. Work by the Campbell laboratory further identified an increase in GABAergic innervation to GnRH neurons in PNA mice that occurs before puberty and with the establishment of hyperandrogenism, but intriguingly, the treatment of adult PNA mice with flutamide is sufficient to reverse the phenotype.^56^ This suggests that the rewiring of GABA cells occurs prenatally, but that high testosterone in later life is required to maintain this alteration. To test if GABA activation of GnRH neurons is a sufficient pathogenic mechanism to generate a PCOS‐like phenotype, the group performed chronic activation of ARC GABA cells, which induced hyperandrogenemia, acylicity, and altered ovarian morphology.^57^

### 3.2 Glial cells

Microglia refine neural connections via “pruning” unnecessary synapses during brain development. As reviewed in detail elsewhere,^58^ changes in microglial regulation of GABAergic synapses to GnRH neurons have been postulated as a mechanism for elevated GABA innervation to GnRH neurons in PCOS mouse models. The authors identified a reduction in microglia and impaired phagocytic pruning of GABA inputs before puberty, potentially resulting in the elevated excitatory innervation to GnRH neurons.^59^ In addition, changes in glial ensheathing of GnRH axon terminals have been implicated in altering GnRH release in PCOS. In a recent study, magnetic resonance spectroscopy and diffusion tensor imaging were used to examine brain structure and function in PCOS patients.^60^ The study found evidence of increased activity in the hypothalamus and increased connectivity between the arcuate nucleus and median eminence compared to controls. Current imaging techniques make it difficult to pinpoint the exact changes in hypothalamic cells in patients. However, the group identified that elevated AMH in the serum of PAMH mice reduces the envelopment of GnRH axon terminals by tanycytes (Figure 2C); specialized glial cells that line the wall of the third ventricle. As tanycyte‐GnRH interactions prevent the diffusion of GnRH to the pituitary portal vasculature, these findings provide a mechanism whereby AMH induces changes in the cytoskeletal structure of tanycytes to result in elevated GnRH/LH release.^60^

### 3.3 KNDy cells

Mutations in the gene encoding the kisspeptin receptor, GPR54 (aka KISS1R), whether occurring globally or specifically within GnRH neurons, lead to hypogonadotropic hypogonadism in both humans and mice.^61^, ^62^ The results of multi‐label immunofluorescence studies in the arcuate nucleus of ewes, goats, and rodents identified kisspeptin neurons in this region that demonstrate a high degree of localization with the tachykinin neurokinin B (NKB) and the endogenous opioid dynorphin. Based on this unique colocalization, this subpopulation of kisspeptin neurons is referred to as KNDy (Kisspeptin, NKB, and Dynorphin) cells.^63^ Functional evidence for the ability of KNDy cells to generate a GnRH/LH pulse includes cell‐specific activation using brief optogenetic stimulation, which is sufficient to generate an LH pulse in mice.^64^ Conversely, optogenetic inhibition of endogenous KNDy cell activity reduces the frequency and amplitude of LH pulses,^65^ and the genetic reduction of ARC kisspeptin levels eliminates LH pulses, estrous cycles, and fertility.^66^ This is comparable with recent findings in sheep, where a reduction in LH pulse amplitude and frequency was seen after lesioning 90% of the KNDy cell population.^67^ The generation of LH pulses by KNDy cells is hypothesized to occur via reciprocal connections within the population. Briefly, this hypothesis suggests that NKB directly promotes glutamate‐driven synchronization of firing among reciprocally connected KNDy cells and initiates the beginning of a pulse. Kisspeptin then serves as an output signal to GnRH neurons, stimulating their activity, while dynorphin acts within the KNDy network as a signal to stop synchronization, thereby ending GnRH/LH release.^63^ The implantation of gradient refractive index (GRIN) lenses coupled with miniature microscopy to record intracellular calcium levels in individual KNDy cells of mice visualized synchronized episodes of activity immediately prior to a pulse of LH release.^68^, ^69^ Therefore, a plethora of evidence supports that the coordination of synchronized episodes of KNDy cell activity is responsible for the generation of GnRH/LH pulses, and therefore, changes in the regulation of KNDy cells may lead to elevated pulsatility. Although it is not technically feasible to study the in vivo activity of KNDy cells in human patients, evidence for serum kisspeptin directly correlating with LH pulses potentially supports that peripheral concentrations may reflect kisspeptin‐GnRH signaling.^70^ In oligomenorrheic PCOS patients, the relationship between kisspeptin and LH pulses is uncoupled, potentially identifying a loss of GnRH pulse regulation by kisspeptin‐containing cells.^71^ Further, a small cohort study identified that serum kisspeptin is higher in infertile PCOS patients compared to fertile PCOS patients, suggesting it may be useful as a diagnostic marker.^72^

In a peripubertal letrozole‐induced mouse model of PCOS, dramatic elevations in ARC kisspeptin and NKB expression are correlated with elevated LH pulse frequency,^73^ and targeted inhibition of KNDy cells using chemogenetic techniques is sufficient to reduce both LH pulses and testosterone levels.^74^ This suggests that elevated KNDy cell activity may underlie LH hypersecretion. The firing of KNDy cells in brain slices from PNA mice is not altered compared to controls,^75^ although synchronized firing of cells, such as those recorded in vivo, has not yet been replicated in sliced mouse brains. In vivo, recordings of KNDy cells in freely behaving PNA mice using fiber photometry identified cyclical changes in KNDy cell episodic activity, despite the mice remaining acyclic according to vaginal smears.^76^ However, in an AI‐identified “diestrus” stage identified by GnRH pulse generator activity, the frequency of synchronized episodes (SE) was significantly elevated compared to controls.^76^ Further, the group found that exogenously delivered progesterone, although effective at reducing SEs in controls, could not lower SEs in PNA mice. Together, these data support the hypothesis that negative feedback inhibition of KNDy cell activity is impaired in PNA mice.

Whether changes in the ability of progesterone to suppress SEs occur directly at KNDy cells or indirectly via upstream populations is unknown. Expression of the nuclear androgen and progesterone receptor in KNDy cells is increased and reduced in PNA mice, respectively,^77^ suggesting a mechanism by which androgen signaling impairs progesterone negative feedback on pulse generation occurs. However, this has not been found by every investigator.^78^ Further, the conditional genetic deletion of progesterone receptors from kisspeptin cells in female mice fails to impair negative feedback regulation of LH pulses,^79^ suggesting that mechanisms beyond reduced progesterone receptor expression are required to impair GnRH pulse regulation. In line with this, extensive rewiring of KNDy neurons and upstream networks has been identified in PCOS animal models. In PNT sheep, KNDy cells form fewer synapses with the GnRH cell body and reciprocal connections between KNDy cells are reduced.^80^ Although not functionally tested, changes in connections between KNDy cells may disrupt steroid hormone feedback from progesterone, which is postulated to occur through dynorphin release at KNDy–KNDy connections. Alternatively, it could represent changes in the coordination of KNDy cell synchronous activity that results in disordered pulses. KNDy cells in PNT sheep also receive a higher density of synaptic input by GABA cells,^55^ although, in PNA mice, a reduction in GABAergic input was detected instead.^55^ Rabies‐mediated tract tracing from KNDy cells in PNA mice revealed widespread changes in synaptic input from other hypothalamic populations^55^ (Figure 2B). Although the precise phenotype of these cells is yet to be identified, they may represent cells relaying internal and external cues to KNDy cells that regulate the tempo of GnRH pulse generation. It is unclear whether changes within KNDy circuitry and upstream networks are reversible; however, encouragingly, treatment with the androgen receptor antagonist Flutamide reduces GABAergic innervation to GnRH neurons in PNA mice.^56^ It is possible that once the anti‐androgen is stopped, the changes in neural circuitry observed in animal models return to pretreatment conditions. This would mimic results in the clinic, in which treatment for hirsutism and other PCOS symptoms using metformin, flutamide, and spironolactone rapidly reappears following the discontinuation of treatment.

### 3.4 Deletion of androgen receptors from genetically defined cell populations to delineate the site of androgen action in PCOS models

In order to delineate the sites of androgen action that are critical for the development of PCOS reproductive, metabolic, and neuroendocrine pathologies, multiple groups have employed cell type‐specific androgen receptor knockout approaches. In a seminal study, the Walters group identified that androgen signaling in the brain, but not the ovary, was critical for the development of reproductive and metabolic symptoms in mice chronically exposed to DHT postnatally.^81^ Subsequently, the Campbell group conditionally knocked out AR from GABA cells in both postnatal DHT and PNA models, but found that this did not prevent the development of reproductive dysfunction.^82^ These data imply that androgenic action at GABA cells is not a critical pathogenic determinant of PCOS. Intriguingly, the group found that elevated GABA input to GnRH neurons in PNA mice was prevented by androgen receptor deletion from GABA cells. This suggests that, while androgen action elsewhere in the brain may suffice to drive PCOS symptoms, ongoing androgen signaling at GABA cells is necessary to maintain abnormal wiring within the GnRH neuronal circuit network. This work also implies that changes in microglia‐mediated pruning of GABA inputs to GnRH neurons do not result from androgen action at glial cells.^82^ In contrast, a study from the Mellon group conditionally deleting androgen receptors from kisspeptin cells in PAMH mice successfully prevented the development of acylicity and infertility, demonstrating that androgen signaling at kisspeptin cells is critical to the development of reproductive traits.^83^ Additional studies are required to pinpoint the specific population of kisspeptin cells responsible for the rescue of reproductive traits, whether in the ovary or within the brain, and to ascertain whether this rescue takes place in utero or during adulthood. The effect of androgen receptor deletion from kisspeptin cells on neuroendocrine parameters in PAMH mice was only evaluated at late adulthood in this study. However, at this age, LH and testosterone levels in PAMH mice are either comparable to or reduced from prenatal vehicle‐treated controls. Therefore, additional research into the role of androgen signaling in kisspeptin neurons related to the development of GnRH/LH hypersecretion and hyperandrogenemia in PCOS models is required.

## 4 TREATMENTS TARGETING ABNORMAL GnRH PULSE GENERATION

Current pharmacological therapies for PCOS treat the pertinent symptoms in a non‐holistic manner. Typically, these include oral contraceptives that aim to improve irregular menstrual cycles, hirsutism, and acne. Anti‐androgens reduce hirsutism, whereas metformin, letrozole, or clomifene promote ovulation. However, no current pharmaceutics can treat the underlying pathophysiology. GnRH receptor antagonism has been utilized in fertility treatments to decrease GnRH‐mediated release of LH and FSH in controlled ovarian stimulation. Aside from fertility treatments, there is limited clinical evidence supporting the use of GnRH antagonists to attenuate GnRH hypersecretion for PCOS therapy. However, recent studies in PAMH mice have shown that intermittent treatment with the GnRH receptor antagonist cetrorelix can reduce testosterone levels, restore oestrous cycles, improve antral follicle development, and increase ovulation.^27^ These data support the hypothesis that the brain is a critical component in PCOS pathogenesis and highlight that treatments targeting GnRH pulsatile secretion may be an attractive strategy for restoring ovulation and fertility in women with LH hypersecretion.

Other novel targets lie upstream of GnRH neurons. One promising population is KNDy cells, for which there has been remarkably rapid progress between discovering the colocalized phenotype in animal models and developing drugs that target KNDy peptide receptors for use in the clinic. Encouraging results have been seen using NKB signaling blockade as a therapeutic agent to target the central pathophysiology of LH hypersecretion and hyperandrogenism in PCOS. The antagonist MLE4901, which has a high affinity for NK3R, the receptor for neurokinin B that is highly expressed in the human brain and within KNDy cells themselves, when administered for at least 7 days, has successfully reduced LH pulsatile secretion in PCOS patients.^84^, ^85^ This reduction was accompanied by decreased total and free testosterone.^84^ In a succeeding study using 12 weeks of treatment with the NK3R antagonist Fezolinetant, which has recently received FDA approval for the treatment of hot flushes, the treatment was also successful at reducing the LH: FSH ratio and suppressing hyperandrogenism in PCOS patients.^86^ However, no changes in ovarian cycles and ovulation were observed over the study's time frame. Potentially, excess endocrine suppression may prevent ovulation with this treatment. However, the authors note that positive clinical outcomes are typically detected in PCOS studies after 6–9 months of treatment. As AMH and ovary size are reduced in this trial, this may be an early indicator of a positive clinical outcome with longer treatment. Further, the reduction in AMH may have beneficial effects on GnRH pulsatility. Therefore, although effective at preventing hyperandrogenemia, additional trials with different lengths and doses of Fezolinetant are still required to determine the outcome of this treatment on restoring spontaneous ovulation. Careful use of the drug will be required to ensure that LH pulsatility, as well as other features such as baseline levels of LH and FSH, are not excessively suppressed when used over a more extended treatment timeframe.

## 5 CONCLUSIONS AND FUTURE CHALLENGES

Increased GnRH pulsatility underpins reproductive and endocrine dysfunction in PCOS. To date, the neural changes that drive GnRH/LH hypersecretion in PCOS patients are unclear, and treatment only targets symptomology and not pathophysiology. Research using animal models has driven significant progress in identifying the specific brain cell populations and neuropeptides that regulate GnRH release and understanding the mechanisms behind changes in pulsatility. This has resulted in the creation of therapeutics targeting KNDy cell signaling to reduce the generation of GnRH/LH pulses in PCOS patients. Yet, our understanding of the PCOS brain and the effect of abnormal central, pituitary, and gonadal hormone release on broader central circuits remains significantly incomplete. For instance, recent studies have highlighted that GnRH may play a vital role in higher‐order processes such as cognition.^87^ Although unstudied, it is possible that central GnRH oversecretion may be linked to cognitive symptoms in PCOS patients.^88^ Animal models will continue to be valuable tools for understanding the mechanistic changes in neural circuitry due to genetic or environmental alterations resulting in PCOS characteristics and associated morbidities. To date, much of the research into mechanistic alterations in the brain that drive PCOS symptomology has been limited to rodents and ruminants. However, it will also be necessary to replicate and extend these findings using non‐human primate models induced via prenatal testosterone treatment or displaying natural hyperandrogenemia.^89^

Another challenge in treating PCOS is the lack of existing therapies that can provide a permanent cure. Preventing the development of PCOS offers the best hope for treatment, but there are obstacles. While it has been suggested that PCOS may have a prenatal origin, there are limited treatment options during pregnancy due to the risk of affecting male fetuses or causing miscarriages. Therefore, identifying biomarkers that can predict the transmission of PCOS from mothers to daughters and foresee changes in neuroendocrine circuits may help identify those at risk of developing elevated GnRH/LH pulsatility. These markers may be combined with treatments that target affected hypothalamic circuits in PCOS to prevent the establishment of GnRH hypersecretion and hyperandrogenemia. Further clinical and preclinical research will be required to characterize the full complement of central circuits that are altered in PCOS.

## AUTHOR CONTRIBUTIONS

**Aleisha M. Moore:** Conceptualization; investigation; funding acquisition; writing – original draft; writing – review and editing.

## FUNDING INFORMATION

This publication was supported by the Eunice Kennedy Shriver National Institute of Child Health and Human Development of the National Institutes of Health under Award Numbers R00HD096120 and R01HD114701 to Aleisha M. Moore.

## CONFLICT OF INTEREST STATEMENT

The author declares no conflicts of interest.

## PEER REVIEW

The peer review history for this article is available at https://www.webofscience.com/api/gateway/wos/peer‐review/10.1111/jne.70028.

## Data Availability

Data sharing is not applicable to this article as no new data were created or analyzed in this study.
